# Therapeutic Potential of Fungal Polysaccharides in Gut Microbiota Regulation: Implications for Diabetes, Neurodegeneration, and Oncology

**DOI:** 10.3390/jof10060394

**Published:** 2024-05-31

**Authors:** Alexandru Stefan Barcan, Rares Andrei Barcan, Emanuel Vamanu

**Affiliations:** 1 Faculty of Biotechnology, University of Agricultural Sciences and Veterinary Medicine, 011464 Bucharest, Romania; 2 School of Biodiversity, One Health & Veterinary Medicine, University of Glasgow, Graham Kerr Building, Glasgow G12 8QQ, UK; 3 Department of Physics, University of Strathclyde, Glasgow G4 0NG, UK

**Keywords:** fungal polysaccharides, gut microbiota, health implications, diabetes, neurodegenerative diseases, cancer, natural compounds

## Abstract

This review evaluates the therapeutic effects of polysaccharides derived from mushroom species that have medicinal and edible properties. The fungal polysaccharides were recently studied, focusing on their modulation of the gut microbiota and their impact on various diseases. The study covers both clinical and preclinical studies, detailing the results and highlighting the significant influence of these polysaccharides on gut microbiota modulation. It discusses the potential health benefits derived from incorporating these polysaccharides into the diet for managing chronic diseases such as diabetes, neurodegenerative disorders, and cancer. Furthermore, the review emphasizes the interaction between fungal polysaccharides and the gut microbiota, underscoring their role in modulating the gut microbial community. It presents a systematic analysis of the findings, demonstrating the substantial impact of fungal polysaccharides on gut microbiota composition and function, which may contribute to their therapeutic effects in various chronic conditions. We conclude that the modulation of the gut microbiota by these polysaccharides may play a crucial role in mediating their therapeutic effects, offering a promising avenue for further research and potential applications in disease prevention and treatment.

## 1. Introduction

The growing prevalence of metabolic diseases, neurodegenerative disorders, and cancer globally presents substantial challenges to health systems, necessitating innovative approaches for prevention and treatment. Functional fungal polysaccharides (FFPs) from both edible and medicinal mushrooms represent a promising field of study due to their health-promoting properties. This review specifically focuses on FFPs derived from well-recognized species such as Shiitake (*Lentinula edodes*), Oyster (*Pleurotus ostreatus*), Reishi (*Ganoderma lucidum*), and Lion’s Mane (*Hericium Erinaceus*) and includes a detailed examination of lesser discussed yet significant species, like *Boletus edulis*, all of which are cataloged in Table 1, along with the associated benefits elucidated therein. Each species was selected based on its unique bioactive compounds and documented benefits ranging from antioxidative to anti-inflammatory effects, with a particular focus on their ability to modulate gut microbiota (GM) [1].

Apart from immediate effects, FFPs exert significant influence through the modulation of the human GM. GM, a complex community of microorganisms residing in the digestive tract, aid in maintaining human health and fight diseases. They are involved in digestion, synthesis of vitamins, immune system development, and protection against pathogens. For instance, in the case of metabolic diseases such as obesity and diabetes, FFPs can enhance glucose regulation and lipid metabolism by inducing measurable but limited alterations in the composition and functional capabilities of the gut microbiota. These alterations, while significant, do not encompass the entire spectrum of possible microbial changes but have been documented to include increases in specific beneficial microbes and decreases in certain detrimental ones [43].

In conditions like Alzheimer’s disease and Parkinson’s disease, which affect the brain, the connection between the gut and the brain becomes very important. This connection, known as the gut–brain axis, involves messages that travel both ways between the gut and the brain [44]. An imbalance in the GM can contribute to inflammation and oxidative stress in the brain, which are linked to the development of these neurodegenerative diseases. FFPs from mushrooms can alter the gut microbiota, potentially mitigating neuroinflammation and oxidative stress, thereby benefiting neurons and slowing the progression of related diseases.

Regarding cancer prevention and treatment, FFPs can not only reduce tumor growth but also serve as a valuable source of nutrients that promote the colonization of beneficial bacteria in the gut [45]. Importantly, even before reaching the colon, FFPs can directly interact with immune receptors in the small intestine, enhancing the body’s protection against cancer cells through a dual mechanism that influences both local and systemic immune responses [46].

To ensure a comprehensive and unbiased review, a systematic search strategy was implemented using databases such as PubMed, Scopus, and Web of Science. Keywords related to ‘fungal polysaccharides’, ‘mushroom health benefits’, and ‘gut microbiota modulation’ were used in combinations to gather the relevant literature from the past decade. Inclusion criteria were defined to select peer-reviewed studies that specifically explored the therapeutic effects of fungal polysaccharides in the context of diabetic mellitus (DM), NDs, and cancer. It also explores the various processes by which these natural substances might help health and disease management, excluding studies not available in English or those lacking empirical data [47].

## 2. Bacterial Commensals

**Figure 1:**
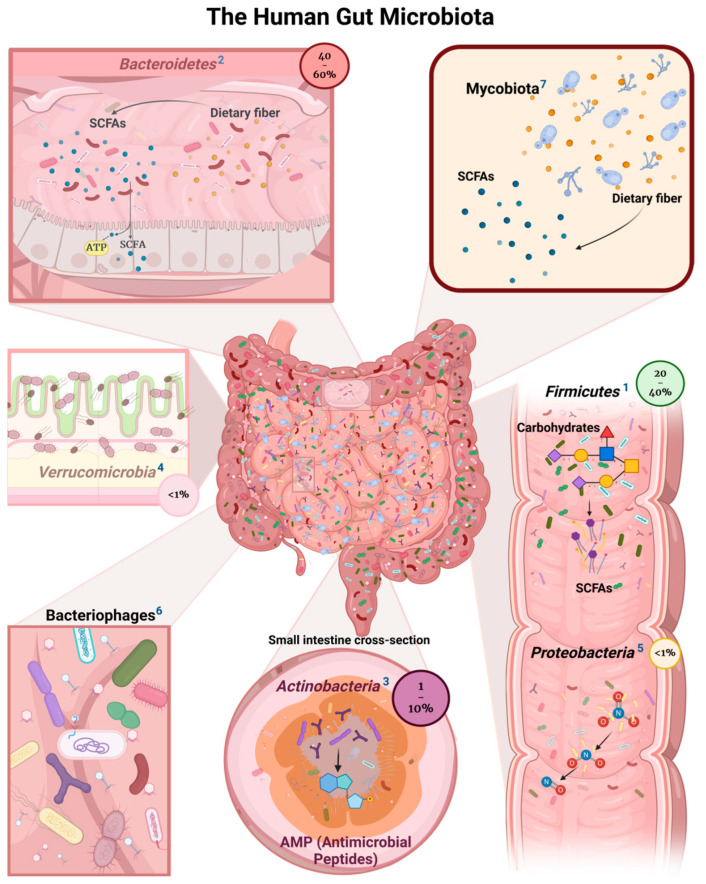
The Human Gut Microbiota. ^1^ Firmicutes are one of the most common types of phyla in GM. They break down complex carbohydrates to produce SCFAs (short-chain fatty acids) like butyrate, acetate, and propionate. These SCFAs are important sources of energy for both the host and the bacteria. Aside from that, Firmicutes produce vitamins, which makes them a good ally of the digestive system [49,50,51]. Among the *Firmicutes*, the family *Lactobacillaceae* is particularly adept at fermenting sugars into lactic acid, a process crucial for maintaining a healthy gut environment. Notably, the genus *Lactobacillus* has been reclassified into 25 distinct genera based on whole-genome sequencing by Zheng et al. (2020), which underscores the genetic and functional diversity within this bacterial group. This reclassification enhances our understanding of the specific roles these bacteria play in the gut, from producing short-chain fatty acids to interacting with the host’s immune system [52]. ^2^ *Bacteroidetes* is a major phylum of Gram-negative bacteria found abundantly in the human gut microbiota. This group of bacteria plays an important role in digesting complex molecules like polysaccharides and fibers, thereby contributing significantly to the host’s energy metabolism and overall gut health. *Bacteroidetes* are involved in breaking down carbohydrates that are indigestible by human enzymes, converting them into SCFAs like acetate, propionate, and butyrate, which serve as energy sources for the host and help maintain the health of the gut lining [53,54]. ^3^ *Actinobacteria*, although not as abundant as *Firmicutes* and *Bacteroidetes*, are critical in the development of immune system processes. They affect gut-associated lymphoid tissue, which is important for immunological maturation and defense against pathogens [55,56]. ^4^ The phylum *Verrucomicrobia* are known for their unique shapes and the ability to degrade various polysaccharides, making them significant in the carbon cycle of their respective habitats [57,58]. ^5^ *Proteobacteria* are typically present at low levels and contribute to the microbial diversity and balance within the gut and take part in various metabolic processes, including the breakdown of complex compounds and the production of substances that can be beneficial for the host’s health [59]. ^6^ Bacteriophages, often referred to as phages, are viruses that specifically infect bacteria. In the human gut, these microscopic entities play a critical role in shaping the composition and functionality of the microbiota [60,61]. ^7^ The mycobiota in the human gut has received relatively less attention compared to its bacterial counterparts, but it plays a significant role in human health and disease control. The gut mycobiota interact with the host’s immune system and other microbes in the microbiome and contribute to the overall balance and functionality of the gut ecosystem [53,62].

Bacteria, as the most abundant elements of the gut microbiota (GM), play a crucial role in promoting and maintaining human health and well-being. The microbial community comprises over 1000 species, with bacteria like *Firmicutes* and *Bacteroidetes* forming 90% of the total microorganisms. However, this community also includes fungi, protozoa, phages, and viruses. Although bacteria are predominant, the specific roles and interactions of these different kingdoms within the gut are not fully understood, highlighting a significant area for further research [48,49] (see Figure 1).

## 3. Fungal Polysaccharides

**Figure 2:**
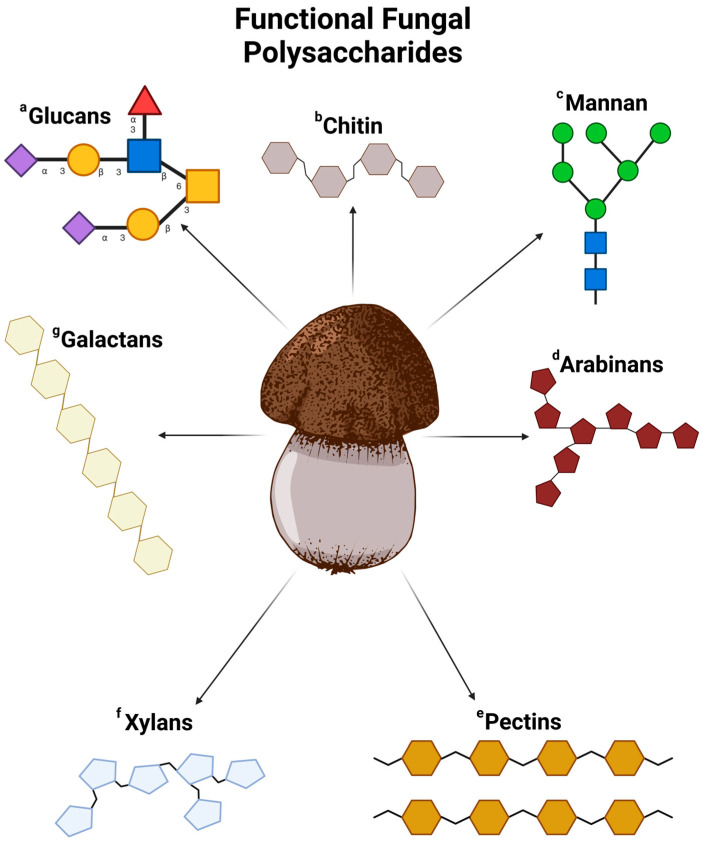
Functional fungal polysaccharides. ^a^ Beta-glucans, found in many fungi, consist of glucose units linked by β-glycosidic bonds, with variations in branching patterns and molecular weights across different species. These polysaccharides also form part of the structural components in fungal cell walls, contributing to the fungi’s structural integrity [70]. In addition to beta-glucans, mushrooms may contain alpha-glucans and other glucans, which have immune-stimulating effects [71]. ^b^ Chitin, a linear polysaccharide made of N-acetylglucosamine units linked by β-(1→4) glycosidic bonds, is a key structural element in fungal cell walls, providing rigidity and protection. Chitin plays various roles in the development of fungi, including growth, cell division, and environmental stress resistance [72]. ^c^ Mannans, a type of hemicellulose in some mushroom cell walls, consist of mannose sugars and have structural and immunomodulatory effects [73]. ^d^ Arabinans, consisting of arabinose sugars, are found in certain mushrooms and may act as dietary fiber [74]. ^e^ Pectins, present in some mushrooms, are complex polysaccharides with galacturonic acid, galactose, and arabinose, known for their gel-forming and thickening properties in food applications [75]. ^f^ Xylans, another hemicellulose found in mushrooms, are made of xylose sugars and may have prebiotic properties that promote beneficial gut bacteria growth [76]. ^g^ Galactans in mushrooms are made of galactose sugars and offer potential health benefits [77].

Diet significantly influences the composition and functions of the gastrointestinal microbiota by affecting the distribution of nutrients among microbial communities. This impact is essential for maintaining a healthy intestinal microbiome and preventing noncommunicable diseases [54,63]. Polysaccharides in mushrooms, especially those in edible and medicinal varieties, are noted for their health benefits. These complex carbohydrates vary widely and are central to the beneficial effects of mushrooms. The health benefits of mushroom polysaccharides are mainly due to their ability to modulate the immune system [64]. They can activate immune cells, improve the body’s infection-fighting capabilities, and have anti-tumor properties, making mushrooms a beneficial addition to the diet for enhancing immune function [65]. Furthermore, fungal fiber polysaccharides, such as chitin and chitosan, provide cardiovascular benefits by helping to lower cholesterol levels and supporting heart health [66,67]. The antioxidant effects of some fungal polysaccharides also play a role in fighting oxidative stress and chronic inflammation, contributing to the prevention of chronic diseases [56,68]. Additionally, the dietary fiber in mushrooms, including these polysaccharides, promotes digestive health by supporting the growth of beneficial gut bacteria and aiding digestion. Understanding the various polysaccharides in fungi and their effects on gut bacteria is important for recognizing their impact on human health [60,69] (Figure 2).

## 4. Bacterial Role in Fungal Polysaccharide Breakdown

Recent studies highlight that various factors, such as diet, living environment, age, sex, health conditions, and medications, significantly shape the composition and functionality of the gut microbiota [78]. Among these, diet influences the gut microbiota’s composition, diversity, and richness [79]; for example, a diet low in carbohydrates leads to a marked decrease in the population of fiber-digesting bacteria like *Ruminoccocus*, *Eubacterium*, *Clostridium*, and *Bifidobacterium* [80]. On the other hand, ingesting polysaccharides from *G. lucidum* and *Poria cocos* can increase the abundance of bacteria capable of degrading xylan and other polysaccharides, such as *Paraprevotella clara* and *Bacteroides xylanolyticus* [81].

Extensive experimental research, both in vitro and in vivo, supports the role of mushroom polysaccharides in regulating the GM. For instance, Su et al. (2019) observed that polysaccharides from *F. velutipes* modified the gut microbiota composition, increasing *Bifidobacteriaceae* and *Bacteroidaceae* while decreasing *Lachnospiraceae* and *Enterococcaceae* populations in fermentation tests [82]. Similarly, Zhao and his collaborators (2019) found that *A. auricular polysaccharides* significantly lowered the *Firmicutes*/*Bacteroidetes* ratio and enhanced fecal microbiota diversity in mice [83]. Additionally, Xu and Zhang (2015) reported that *L. edodes* polysaccharides also reduced the *Firmicutes*/*Bacteroidetes* ratio but decreased overall microbiota diversity and evenness, particularly in the cecum and colon [84].

The varying outcomes observed with different polysaccharides can be attributed to their distinct structures and hydrolysis mechanisms [85]. The specific glycosidic linkages and molecular sizes of these polysaccharides are thought to influence the profiles of intestinal microbial communities significantly [86]. Studies using animal models have demonstrated that consumption of edible fungal polysaccharides (EFPs) can significantly modify the composition and diversity of the gut microbiota (GM). Research involving pigs showed that Lentinan could mitigate intestinal harm caused by lipopolysaccharide (LPS) through adjustments in the GM’s structure and metabolites [87,88,89]. In models of inflammation induced by dextran sulfate sodium (DSS), administration of 50 mg/kg of *Flammulina velutipes* polysaccharides improved the gut’s microbial balance. This was achieved by boosting populations of bacteria that produce SCFAs such as *Ruminal butyrivibrios* and *Roseburia* while decreasing pathogenic bacteria. Furthermore, a dose of 100 mg/kg of these polysaccharides increased the levels of the *Bacteroidales* family S24-7, which has been shown to alleviate colitis symptoms [90]. In models of diet-induced obesity, polysaccharides from *Sarcodon aspratus* lowered the ratio of *Firmicutes* to *Bacteroidetes* and enhanced the presence of beneficial microbes, including *Lactobacillus*, *Akkermansia*, and *Bacteroides* [34]. A similar reduction in the *Firmicutes*/*Bacteroidetes* ratio was observed in mice that were fed polysaccharides derived from *Auricularia auricular* [83].

## 5. Beta-Glucans and Their Impact on Gut Microbiota

The health impacts related to beta-glucans have been highly investigated, making them one of the most explored polysaccharides [91]. This section deals with an in-depth analysis of beta-glucans, a non-digestible carbohydrate that supports colonic microbial growth. The small intestine does not absorb it, leaving it undigested as it travels through various parts of the digestive tract where it interacts with the microbiota. Beta-glucan has been found to stimulate an increase in the numbers of beneficial bacteria such as *Lactobacillus casei*, *Bifidobacterium animalis* subsp. *lactis*, and *Lactobacillus acidophilus* in both laboratory and natural conditions [92]. When mice were fed cereal beta-glucan, more *Bifidobacteria* and *Lactobacilli* strains were observed, among others [93]. Also, diets containing whole grain barley pasta and durum wheat flour, which are rich in beta-glucan, showed a higher population of specific types of bacteria like *Clostridiaceae*, e.g., *Roseburia hominis*, *Ruminococcus* sp., and *Clostridium orbiscindens* (*Clostridium* sp.), among others, while a lesser population of *Firmicutes* and *Fusobacteria* was observed [94]. Yogurt supplemented with oat or barley beta-glucans promoted increased growth rates and viability of *Bifidobacterium animalis* subsp. *Lactis* [95].

The diversity in the gut bacteria’s ability to digest dietary glycans is notable. *Bifidobacterium*, for example, cannot digest complex glycans such as pectin and depends on *Bacteroides* to break down pectin into oligosaccharides before it can utilize them [96]. This relationship forms a syntrophic system where different bacteria evolve to maintain a balance in the gut microbiota. In the gut microbiota, *Bifidobacterium* and *Bacteroides* are necessary, making up about 80% of the microbial community in both infants and adults, and play essential roles in metabolizing dietary glycans [97]. *Bacteroides* act as primary degraders by breaking down complex glycans, while *Bifidobacterium* functions as secondary degraders for simpler glycans [98].

## 6. Human Gut Bacteria’s Affinity for β-Glucan

Gut *Bacteroidetes* have the capability to detect and respond to a range of glycans in their environment, a process supported by the various extracellular sensor-regulator systems connected to the polysaccharide utilization loci (PUL) that they possess [99]. The hybrid two-component system (HTCS) is the most thoroughly studied of these systems, both biochemically and structurally [100]. The HTCS consists of a single protein that extends across the cytoplasmic membrane and includes domains found in a typical two-component system: an extracellular sensor at the N-terminus, a cytoplasmic histidine kinase, and a response regulator [101]. The HTCS detects signals through the direct attachment of oligosaccharide fragments to the periplasmic sensor domain [102]. These oligosaccharides, results of polysaccharide breakdown by endo-acting enzymes encoded within the PUL anchored to the cell surface, are first moved into the periplasm by the SusC homolog, a TonB-dependent transporter. Sometimes, these oligosaccharides are further processed by periplasmic enzymes before acting as activation signals [103].

In comparison, the sensing mechanism in Gram-positive bacteria, such as *Bifidobacterium*, is less understood. However, known transporters, including those with highly conserved sequences in the solute binding protein, facilitate the uptake of glucans [104]. *Bifidobacterium animalis* subsp. *lactis*, for instance, has developed an ABC transport system for the uptake of complex glycans like arabinoxylan [105]. The solute-binding protein within this system can efficiently transport arabinoxylan-derived oligosaccharides, showing a particular adaptability for tri-saccharides and tetra-saccharides, including those with xylo- and arabinose decorations [106]. Structural studies indicate that this protein has a large binding pocket and a flexible lid-like loop that aids in binding various decorated oligosaccharides. This protein’s widespread presence across the *Bifidobacterium* genus highlights its importance in metabolic processes within the gut microbiota and interactions with other microbial species [107].

Less information is available on how *Bifidobacterium* processes glycans compared to *Bacteroides*. Given the significant role of *Bifidobacterium* in probiotics, understanding the genes and enzymes that allow Gram-positive bacteria to identify, break down, and transport complex glycans is imperative. Discovering new pathways within *Bifidobacterium* could lead to a better comprehension of the metabolites essential for maintaining gut balance [108].

## 7. Impact of Fungal Fiber Polysaccharides on Diabetes Care

FFPs hold substantial promise in offering benefits to individuals living with both T1D and T2D, with their effects centered on modulating GM. These natural compounds, prevalent in mushrooms like Reishi, Shiitake, and Maitake, provide a novel avenue for enhancing the health of those affected by these diabetes types [109]. T1D is an autoimmune condition where the body’s immune system mistakenly targets and destroys insulin-producing cells in the pancreas [110]. The absence of insulin leads to uncontrolled blood sugar levels and common symptoms such as excessive thirst, frequent urination, fatigue, weight loss, and slow wound healing. Management typically involves lifelong insulin injections or insulin pump use, constant monitoring of blood sugar, and vigilance in diet and exercise [111]. In contrast, T2D usually develops due to insulin resistance, where the body’s cells become unresponsive to insulin, and the pancreas struggles to produce enough of this hormone. This type is often associated with obesity and lifestyle factors. Managing T2D often involves oral medications, lifestyle changes, and sometimes insulin [112]. Both diabetes types have a strong link to GM. An imbalance in GM, known as dysbiosis, can lead to insulin resistance and inflammation, exacerbating the challenges faced by individuals with diabetes [113].

FFPs help manage blood sugar by reducing carbohydrate absorption in the intestines, resulting in more stable post-meal blood sugar levels [114]. This effect is particularly important for individuals with type 1 and type 2 diabetes, as consistent blood glucose levels are key to their control [115]. Additionally, these compounds enhance insulin sensitivity, which aids in more effective glucose uptake into cells, a vital aspect of diabetes care [116]. FFPs also act as prebiotics, nourishing beneficial gut bacteria and promoting a diverse and healthy GM [117]. This contributes to better metabolic health, reduced inflammation, and improved glucose regulation. Furthermore, the anti-inflammatory properties of FFPs are beneficial for reducing the chronic inflammation commonly seen in diabetes, thereby aiding in better glucose control [118]. Comparing the two diabetes types, while they differ in their root causes, the impact of FFPs on GM modulation holds potential for benefits in both cases. In T1D, where insulin production is inadequate due to immune system dysfunction, these compounds can aid in managing blood sugar levels and insulin sensitivity [119]. For T2D, marked by insulin resistance often linked to obesity, FFPs can contribute to better glucose control and improved insulin sensitivity [120].

Taylor and Vasu (2021) discovered that oral administration of Yeast β-glucan (YBG) in juvenile NOD mice significantly altered GM, boosting metabolic pathways and immune regulatory cytokines. Notably, YBG-treated mice exhibited delayed T1D onset and reduced insulitis, suggesting its potential for delaying T1D in at-risk individuals [121]. Rehman et al. (2022) explored the impact of *Morchella esculenta* polysaccharide (MEP) on T2D induced in mice by a high-fat diet and streptozotocin. MEP administration effectively mitigates hyperglycemia and hyperlipidemia and enhances insulin sensitivity. GM analysis reveals an increase in beneficial bacteria (*Lactobacillus* and *Firmicutes*) and a decrease in opportunistic bacteria (*Actinobacteria*, *Corynebacterium*, and *Facklamia*). MEP also reduces endotoxemia and pro-inflammatory cytokines, and improves intestinal permeability. Furthermore, MEP influences the metagenome of microbial communities in T2D mice, emphasizing its prebiotic potential in ameliorating the GM and its metabolites in T2D [32].

Research on the impact of *Boletus edulis* on gut microbiota regulation is limited. Prior studies from our laboratory have shown its ability to regulate gut microbiota and mitigate diabetes and antibiotic-induced dysbiosis through its antihyperglycemic and anti-inflammatory effects. These findings highlight the potential for expanding research in this area [42].

## 8. Functional Fungal Polysaccharides and the Gut–Brain Connection in Preventing Neurodegenerative Diseases

Neurodegenerative diseases (NDs), including Alzheimer’s Disease (AD) and dementia, are increasing and becoming a global health concern. Research into functional fungal polysaccharides (FFPs), especially beta-glucans and polysaccharide peptides found in mushrooms such as Lion’s Mane and Reishi, suggests these compounds could play a significant role in managing NDs. These compounds may influence NDs by affecting the gut microbiota (GM) and the gut–brain axis. NDs are marked by the gradual loss of nerve cells, resulting in cognitive decline, memory loss, and reduced motor skills. Conditions like AD and dementia significantly challenge healthcare systems worldwide, prompting the need for new prevention and treatment methods [122]. NDs are also associated with early-stage gastrointestinal issues, such as constipation and delayed gastric emptying, alongside an imbalance in intestinal flora [123].

**Figure 3:**
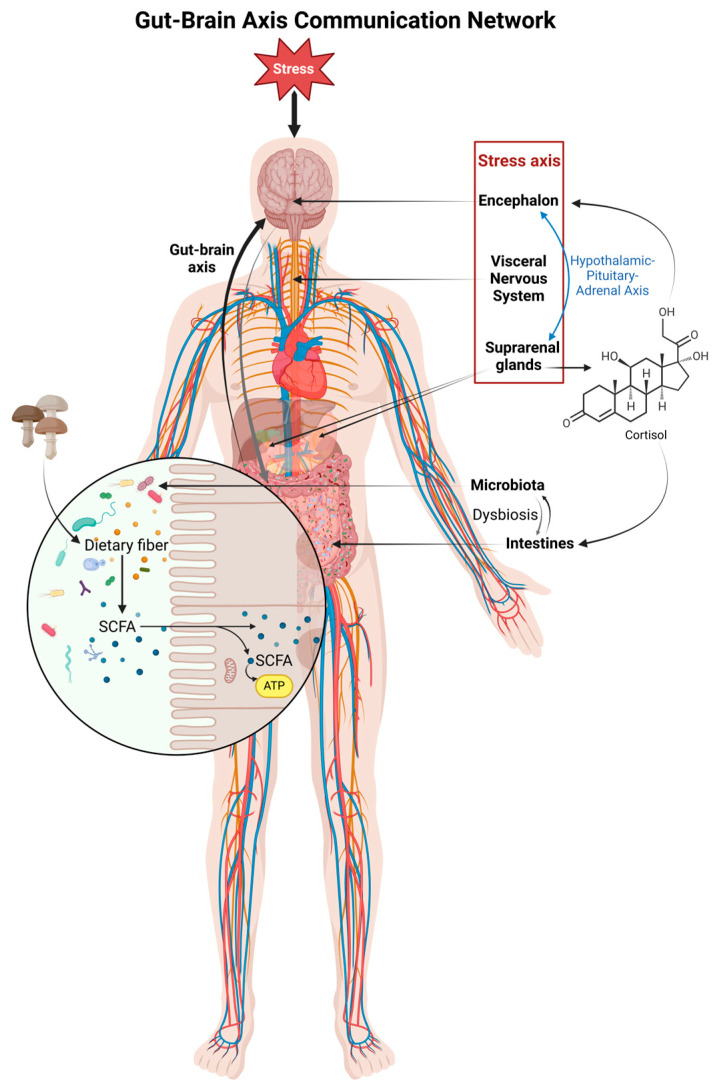
Gut–brain axis communication network.

A recently established and key link between the gut and the brain, the gut–brain axis involves several functions, including its impact on brain function (Figure 3) [124,125]. This connection has been shown in animal models, and there is a noticeable connection between gut bacteria and AD. Indeed, the difference in the abundance of gut bacteria in AD patients is demonstrated through various bacterial case-control studies [126,127]. For example, different levels of the bacterium *Bacteroidetes* and decreases in the levels of the bacterium *Firmicutes* and *Actinobacteria* have been associated with AD biomarkers [128]. Studies from Japan and Turkey have also investigated the influence of GM on cognitive health and speculated on the protective effects of certain bacterial strains from NDs. Thus, distinct gut–brain linkages highlight the scientific reasoning behind the context analysis and offer a new perspective for possible therapeutic approaches [129,130]. Dysbiosis, also known as imbalanced GM, is also involved in the final advance of NDs. The metabolic production of short-chain fatty acids with the metabolites also contributes to their function in neuroimmune, nerve signaling, and brain metabolism [131]. Changes in the bacterial species, such as decreases in beneficial bacteria and increases in proinflammatory bacteria, are engaged in the pathogenesis of AD and dementia [132].

In Figure 3, stress is depicted as a stimulus that impacts the central nervous system (CNS), influencing both the hypothalamic–pituitary–adrenal (HPA) axis and the visceral nervous system (VNS), which governs involuntary physiological processes. The CNS communicates with the gastrointestinal (GI) tract, where trillions of gut microbes reside and interact with the local mucosal environment, as shown in the inset of the colon barrier. The gut microbiota can modulate brain function and metabolism by producing signaling molecules, such as short-chain fatty acids (SCFAs) from the fermentation of dietary fibers, which can enter systemic circulation and reach distant target sites, including the brain.

The CNS’s influence on the GI tract is shown as the dominant force through the ANS and the HPA axis. Conversely, the gut provides feedback to the brain, closing the loop of bidirectional communication. This relationship is pivotal in maintaining homeostasis and is implicated in a range of physiological and psychological processes. The liver is also suggested to play a role in this interaction, with the gut microbiota affecting liver function, which in turn can have systemic effects [124]. FFPs have anti-inflammatory properties that may prevent inflammations associated with NDs. Additionally, they maintain the integrity of the gut–brain connection and impact the production of neuromodulators through gut–brain connectivity. In other words, they aid in preventative psychology and promote cognitive and overall brain well-being [133]. Furthermore, certain mushrooms also contain compounds that can help nerve development [134]. FFPs nourish the gut bacteria, hence facilitating their growth and enhancing the overall well-being of GM [135]. This not only results in improved development and a fortified gut ecosystem but also reinforces the gut barrier [136]. Consequently, the transportation of detrimental compounds that may otherwise have an adverse impact on brain health is diminished. Moreover, GM have a preference for polysaccharides, which help promote the growth of specific microorganisms and their metabolic activities after fermentation [137].

There is evidence that an alteration in the composition of the gut microbiota has an impact on the initiation of AD at the phylum level. For example, there have been observations where mice with AD showed a decrease in *Firmicutes* and an increase in *Bacteroidetes*, possibly due to beta-amyloid accumulation and systemic inflammation in cognitive impairment [138]. The effects of *Polygonatum sibiricum* polysaccharide (PSP) on inflammatory bacteria present in the intestine were also investigated through studies [139]. The findings indicated significant reductions in certain bacteria associated with inflammation, thus reducing neuroinflammatory stress and probably impacting the neurodegenerative disorders’ progression [140].

Han and his collaborators (2019) highlighted polysaccharides from *Inonotus obliquus* and its fruiting bodies, showing their antioxidative and antiapoptotic properties that offer protection against AD [141]. Similarly, polysaccharides from *Amanita caesarea* have been found to have protective effects against AD progression, suggesting mushrooms’ potential role in treating this condition [142].

## 9. Fungal Fiber Polysaccharides and Cancer Treatment

**Figure 4:**
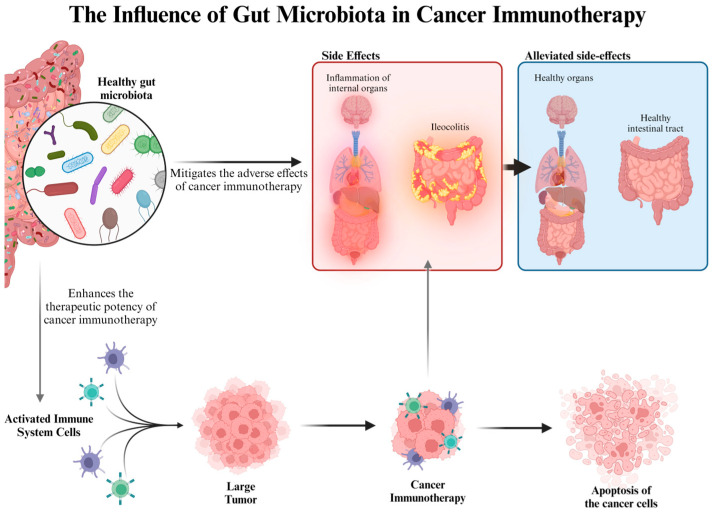
The influence of gut microbiota in cancer immunotherapy.

One of the greatest threats to world health is cancer, a group of diseases characterized by the uncontrolled growth and potential spread of abnormal cells. Based on the search results provided, there is evidence from multiple sources that supports the claim that functional fungal polysaccharides can interact with the immune system and potentially have anticancer effects [143,144] (Figure 4) [145]. Certain bacteria, including *Fusobacterium nucleatum* [146], *Streptococcus gallolyticus* [147], *Enterococcus faecalis* [148], enterotoxigenic *Bacteroides fragilis* [149], and *Escherichia coli* [150], have been implicated in the development and progression of colorectal and other gastrointestinal cancers. These microorganisms are known to influence carcinogenesis through various mechanisms such as promoting inflammation, inducing DNA damage, and suppressing anti-tumor immune responses. Often, an increased abundance of these bacteria correlates with heightened cancer risk. Consequently, we hypothesize that nurturing non-opportunistic pathogenic bacterial populations and suppressing harmful ones may reduce tumor development. Additionally, daily intake of polysaccharides enhances the immune system, potentially aiding in the suppression of certain tumors [143,144]. Furthermore, the byproducts of polysaccharide fermentation, primarily short-chain fatty acids (SCFAs) like butyrate, propionate, and acetate, have direct immune-modulating properties. SCFAs can influence the activity of various immune cells, including macrophages, dendritic cells, and T lymphocytes [151]. Chronic inflammation is a well-known risk factor for several types of cancer, including colorectal cancer. By altering the microbiota and increasing the production of SCFAs, polysaccharides can reduce gut inflammation [152].

Figure 4 presents a schematic overview of the influence of gut microbiota on cancer immunotherapy. It depicts a three-part interaction:

On the left, “Healthy gut microbiota” is shown with a diverse array of bacteria, suggesting a balanced intestinal flora. This healthy microbiome is linked to “Activated Immune System Cells”, indicating that a diverse gut microbiota can prime the immune system to respond more effectively.

In the center, “Cancer Immunotherapy” is shown targeting a “Large Tumor”, illustrating the primary function of immunotherapy. However, this intervention has potential “Side Effects”, demonstrated by an inset showing “Inflammation of internal organs” and specifically “Ileocolitis”, characterized by inflamed intestines.

On the right, the figure outlines the desirable outcome of “Alleviated side-effects”, where the healthy organs and intestinal tract are restored, implying that a healthy gut microbiota can help combat the adverse effects of immunotherapy. Additionally, the “Apoptosis of the cancer cells” indicates the successful targeting of tumor cells by the activated immune system, facilitated by cancer immunotherapy [153].

Some pathogenic gut microbiota promote cancer development through multifactorial action. For example, some bacteria may act directly through DNase activity, while other may act indirectly through producing inhibition metabolites of the DNA repair mechanism. Some specific bacterial species that produce toxins seem to increase the risk of cancer. For example, research results suggest that oncogenesis can be driven by pathogenic microorganisms affecting metabolic pathways, signaling processes, and host immune responses [145,154,155,156]. In their 2019 study, Khan and colleagues explored the impact of saponins from *Gynostemma pentaphyllum* (GpS) and polysaccharides from *Ganoderma lucidum* (GLP) on Apc Min/+ mice. They found significant improvements in the inflamed gut barrier, evidenced by reduced polyps, a shift from M1 to M2 macrophages in the colon, a favorable adjustment in the E-cadherin/N-cadherin ratio, and a decrease in oncogenic signaling molecules. Additionally, the treatments notably increased the presence of SCFA-producing bacteria and reduced sulfate-reducing bacteria in a time-dependent manner [157].

Another study compared the gut microflora of melanoma patients being treated with anti-PD-1 therapy. The authors found differences in the diversity and composition of cancer patients who were responders compared to those who did not respond to treatment [158]. In a separate investigation, researchers also identified several distinct GM species that had good reactions in malignant melanoma (MM) patients undergoing anti-PD-1 immunotherapy. Some of these species included *Bifidobacterium longum* and *Enterococcus faecium*, which have been shown to have anti-tumor responses [159]. Other substances, such as the fractionate β-glucan from *Lentinus edodes*, show promise in inhibiting CRC growth and supporting intestine health [160]. In their study, Zhang et al. (2023) found that *Grifola frondosa* polysaccharide–protein complexes show anti-hepatocellular carcinoma effects and also have an have an effect on GM composition, making *G. frondosa* PPCs a potential natural ingredient for HCC treatment and regulating GM [161].

In addition, the gut microbiota play an integral part in various other illnesses, extending beyond CRC [162]. Studies have started to reveal connections between gut bacteria and various forms of cancer, such as breast, liver, gastric, and intestinal cancers [163,164,165,166]. These findings present new possibilities for the development of targeted cancer treatments and prevention strategies, emphasizing the significant impact of our gut bacteria on our overall well-being [167,168].

## 10. Future Perspectives

This article highlights the significant benefits of FFPs derived from certain mushrooms, specifically in relation to diabetes, neurodegenerative diseases, and cancer. One potential benefit of individual polymicrobial composition is the potential for personalized therapy through the identification of FFPs with the assistance of specific polysaccharide functions. Adopting an individualized therapeutic strategy can enhance therapeutic effectiveness, mitigate adverse effects, and reduce treatment toxicity. Utilizing FFPs can enhance the curative effect and perhaps decrease the required dosage of current medications when used in conjunction with traditional therapy.

Finally, FFPs’ modulating role in GM expands potential preventive health. One could integrate these compounds into a dietary or nutritional plan to lower the risk of diabetes, NDs, or cancer. There is a wide range of other mushroom species that should be explored for their potential to identify and isolate novel compounds, making this area a productive one for further investigation. It may also result in the discovery of new therapeutic agents and lead to a broadening of the spectrum of health applications. Large-scale clinical trials are needed to apply these promising perspectives in a clinical setting. Such efforts will provide conclusive evidence of FFPs’ safety and efficacy and may facilitate their introduction into standard medical treatment.

## 11. Conclusions

The complex relationship among functional fungal polysaccharides, gut microbiota, and human health presents a promising area for advancements in the management of diabetes, neurodegenerative diseases, and cancer. However, the evidence supporting the potential benefits of FFPs in these conditions varies in quality and quantity. In the case of diabetes, several preclinical studies have demonstrated the ability of FFPs to modulate blood glucose levels, insulin sensitivity, and metabolic parameters by altering the gut microbiota composition. These findings suggest that FFPs may have the potential to serve as complementary or alternative therapies for diabetes management. However, the translation of these results to clinical settings requires well-designed, large-scale human trials to establish the efficacy and safety of FFPs in diabetic patients. The neuroprotective effects of FFPs and their potential implications for neurodegenerative diseases, such as Alzheimer’s disease, have been explored in both in vitro and animal models. These studies indicate that FFPs may influence cognitive function by modulating the gut microbiota and mitigating neuroinflammation and oxidative stress. While these results are promising, further research is needed to validate the findings in human subjects and elucidate the specific mechanisms underlying the observed neuroprotective effects. In the context of cancer, preclinical evidence suggests that FFPs may exhibit anti-tumor properties by modulating the gut microbiota and enhancing the body’s immune responses. However, the quality of the available evidence varies, with most studies conducted in cell lines or animal models. Robust clinical trials are necessary to assess the efficacy and safety of FFPs as adjuvant or primary therapies in cancer patients.

## Figures and Tables

**Table 1:** Therapeutic effects of mushroom-derived polysaccharides.

	Study Type	Functions and Health Benefits	References
*Ganoderma lucidum* Polysaccharides	In Vivo, In Vitro	Enhances glucose metabolism, reduces colorectal cancer growth, promotes gut health, and protects against Alzheimer’s.	[2,3,4,5]
*Inonotus obliquus* Polysaccharides	In Vivo, In Vitro	Ameliorates type 2 diabetes, lowers lipids, and inhibits tumor growth in colitis-associated cancer.	[6,7,8]
*Hericium erinaceus* Polysaccharides	In Vivo, In Vitro	Regulates glucose and lipid metabolism, enhances gut and liver health, improves cognitive function in mice.	[9,10,11]
*Lentinula edodes* Polysaccharides	Clinical Trial, In Vivo	Evaluated in subjects with high cholesterol; no lipid change but improved dietary fiber intake and gut microbiota. Protects against diet-induced cognitive decline in mice, enhancing gut–brain axis and synaptic functions.	[12,13,14]
*Auricularia auricula* Polysaccharides	In Vivo	Boosts immune function in treated mice, enhances cytokines and tight junction proteins, and restores gut microbiota, suggesting use in food and pharmaceuticals.	[15,16]
*Grifola frondosa* Polysaccharides	In Vivo	Shows anti-hepatocellular carcinoma effects; modulates gut microbiota and SCFA in diabetic mice, improving intestinal and microbial balance.	[17,18,19,20]
*Pleurotus ostreatus* Polysaccharides	In Vivo	Supports gut health in *A. japonicus* and immunosuppressed mice and enhances immune response, SCFAs, and gut microbiota diversity.	[21,22,23]
*Gynostemma pentaphyllum* Polysaccharides	In Vivo	Modulates gut microbiota, enhances anticancer effects in mice. Reduces oxidative stress and inflammation in diabetic mice.	[24,25,26]
*Amanita caesarea* Polysaccharides	In Vivo	Polysaccharides enhance cognitive function, alleviate inflammation, and modulate oxidative stress in Alzheimer’s models.	[27,28]
*Polygonatum sibiricum* Polysaccharides	In Vivo, In Vitro	Improves memory and cognition in Alzheimer’s mice, enhances SCFAs and LCFAs, and modulates gut microbiota balance.	[29,30,31]
*Morchella esculenta* Polysaccharides	In Vivo, In Vitro	Regulates hyperglycemia and hyperlipidemia, improves insulin sensitivity and gut microbiota, reduces inflammation, and enhances intestinal health.	[32,33]
*Sarcodon aspratus* Polysaccharides	In Vivo	Ameliorates obesity-related metabolic disorders, modulates gut microbiota, enhances probiotics, and reduces stress-triggered bacteria.	[34,35,36]
*Flammulina velutipes* Polysaccharides	In Vivo	Mitigates CdCl2-induced intestinal inflammation and ulcerative colitis in mice, modulates gut microbiota and SCFAs, and enhances metabolic capacity.	[37,38]
*Poria cocos* Polysaccharides	In Vivo	Improves glucose intolerance, insulin resistance, and lipid metabolism. Prevents NASH progression by modulating gut microbiota and suppressing inflammation.	[39,40]
*Boletus edulis* Polysaccharides	In Vitro, In Vivo	Modulates human microbiota, reduces inflammation, enhances antioxidant capacity, and mitigates antibiotic-induced and diabetes-associated dysbiosis.	[41,42]

## Data Availability

Data are contained within the article.

## Abbreviations

AD—Alzheimer’s disease; CRC—Colorectal cancer; FFPs—Functional fungal polysaccharide; GLP—*Ganoderma lucidum* polysaccharide; GM—Gut microbiota; HCC—Hepatocellular carcinoma; HTCS—Hybrid two-component system; MEP—*Morchella esculenta* polysaccharide; ND—neurodegenerative disease; NGF—Nerve growth factor; PPCs—Polysaccharide–protein complexes; PSP—*Polygonatum sibiricum* polysaccharide; PUL—Polysaccharide utilization loci; SCFAs—Short-chain fatty acids; T1D—Type 1 diabetes; T2D—Type 2 diabetes; YBG—Yeast β-glucan.
